# Three-month life expectancy as inclusion criterion for clinical trials in advanced pancreatic cancer: is it really a valid tool for patient selection?

**DOI:** 10.1007/s12094-023-03323-1

**Published:** 2023-10-04

**Authors:** Lena Weiss, Volker Heinemann, Laura E. Fischer, Frank Gieseler, Thomas Hoehler, Julia Mayerle, Detlef Quietzsch, Anke Reinacher-Schick, Michael Schenk, Gernot Seipelt, Jens T. Siveke, Michael Stahl, Ursula Vehling-Kaiser, Dirk T. Waldschmidt, Klara Dorman, Danmei Zhang, C. Benedikt Westphalen, Michael von Bergwelt-Baildon, Stefan Boeck, Michael Haas

**Affiliations:** 1 Department of Medicine III, University Hospital, LMU Munich, Munich, Germany; 2 Comprehensive Cancer Center, University Hospital, LMU Munich, Munich, Germany; 3 German Cancer Consortium (DKTK), Partner Site Munich, Munich, Germany; 4 Department of Hematology and Oncology, University Hospital Schleswig-Holstein-Campus Lübeck, Lübeck, Germany; 5 Department of Medicine I, Prosper Hospital, Recklinghausen, Germany; 6 Department of Medicine II, University Hospital, LMU Munich, Munich, Germany; 7 Department of Medical Oncology, Klinikum Chemnitz, Chemnitz, Germany; 8 Department of Hematology and Oncology, St. Josef Hospital, Ruhr University, Bochum, Germany; 9 Department of Hematology and Oncology, Klinikum Barmherzige Brüder, Regensburg, Germany; 10 Onkologie Main-Taunus, Bad Soden, Germany; 11 West German Cancer Center, Bridge Institute of Experimental Tumor Therapy, University Hospital Essen, University of Duisburg-Essen, Essen, Germany; 12 Department of Medical Oncology, Evang. Kliniken Essen-Mitte, Essen, Germany; 13 MVZ Dr. Vehling-Kaiser GmbH, Landshut, Germany; 14 Department of Gastroenterology and Hepatology, University of Cologne, Cologne, Germany; 15 Division of Solid Tumor Translational Oncology, German Cancer Consortium (DKTK Partner Site Essen) and German Cancer Research Center, DKFZ, Heidelberg, Germany

**Keywords:** Clinical trial, Inclusion criterion, Pancreatic cancer, Prognosis

## Abstract

**Purpose:**

To analyze the 3-month life expectancy rate in pancreatic cancer (PC) patients treated within prospective trials from the German AIO study group.

**Patients and methods:**

A pooled analysis was conducted for patients with advanced PC that were treated within five phase II/III studies conducted between 1997 and 2017 (Gem/Cis, Ro96, RC57, ACCEPT, RASH). The primary goal for the current report was to identify the actual 3-month survival rate, a standard inclusion criterion in oncology trials.

**Results:**

Overall, 912 patients were included, 83% had metastatic and 17% locally advanced PC; the estimated median overall survival (OS) was 7.1 months. Twenty-one percent of the participants survived < 3 months, with a range from 26% in RC57 to 15% in RASH. Significant predictors for not reaching 3-month OS were > 1 previous treatment line (*p* < 0.001) and performance status (*p* < 0.001).

**Conclusions:**

Despite the definition of a life expectancy of > 3 months as a standard inclusion criterion in clinical trials for advanced PC, a significant proportion of study patients does not survive > 3 months.

**Trial registration numbers:**

NCT00440167 (AIO-PK0104), NCT01729481 (RASH), NCT01728818 (ACCEPT).

## Introduction

Pancreatic cancer still remains one of the most lethal malignancies and is projected to become the second leading cause of cancer-related death by 2030 [1]. Numerous clinical trials have been conducted since the introduction of gemcitabine in 1997, with most of them being negative for the primary endpoint OS [1]. Two positive phase III studies (PRODIGE/ACCORD-11/0402 and MPACT) have implemented our current standard regimens for metastatic pancreatic cancer, FOLFIRINOX and gemcitabine/nab-paclitaxel [2, 3]. Our group has conducted five prospective, multicenter phase II/III studies in advanced pancreatic cancer since 1997, all of them within the national AIO (“Arbeitsgemeinschaft Internistische Onkologie”) study group of the German Cancer Society. All five trials (Gem/Cis, Ro96, RC57, ACCEPT, RASH) have been published in peer-reviewed international journals: details on study design and clinical results from each study can be found in these original reports [4–8]. Additionally, the major trial characteristics of the current pooled analysis from these first-line studies are summarized in Table 1.

**Table 1:** Summary of trial characteristics of the included studies for the pooled analysis

Characteristic	All patients	Gem/Cis	Ro96	RC57	ACCEPT	RASH
Number of patients, *n*	912	190	188	274	115	145
Treatment arm		A: Gem/Cis B: Gem	A: Cap/Gem B: Cap/Ox C: Gem/Ox	A: Cap/Erlo-Gem B: Gem/Erlo-Cap	A: Gem/Afa B: Gem	A: Gem/Erlo B: Gem/Erlo-FOLFIRINOX
Phase of trial		III	II	III	II	II
Primary endpoint		OS	PFS after 3 months	TTF after 1st and 2nd line therapy	OS	1-year survival rate in rash-positive patients ≥ 40%
Recruiting period	12/1997–01/2017	12/1997–01/2002	07/2002–06/2004	05/2006–12/2008	04/2013–01/2017	07/2012–07/2015
UICC-Stage	III–IV	III–IV	III–IV	III–IV	IV	IV

*Gem* Gemcitabine, *Cis* Cisplatin, *Cap* Capecitabine, *Ox* Oxaliplatin, *Erlo* Erlotinib, *Afa* Afatinib, *OS* overall survival, *PFS* progression-free survival, *TTF* time to treatment failure

Nearly all clinical trials in gastrointestinal oncology (and in oncology overall) have introduced predefined inclusion criteria regarding life expectancy: in pancreatic cancer usually a life expectancy of at least 3 months is required to enter a patient in a prospective trial investigating systemic therapy. The purpose of this approach usually is to exclude patients with a very poor prognosis (e.g., determined either by host factors or tumor biology), often associated with a risk of early drop-out, and thus allowing a profound analysis of relevant clinical endpoints like objective treatment response and toxicity in the study cohort [9]. However, this specific inclusion criterion of a life expectancy of at least 3 months is of course based on the subjective judgement of the local investigator.

We, thus, aimed to analyze the 3-month survival rate und clinical predictors of this time-to-event endpoint in a large cohort of pancreatic cancer patients who all were treated within trial protocols of the AIO study group conducted during the last two decades in a German multicenter setting.

## Materials and methods

Five prospective, multicenter AIO trials led by our Munich group were included in this pooled analysis: Gem/Cis, Ro96, RC57 (AIO-PK0104), ACCEPT, RASH [4–8]. The overall recruiting period was December 1997–January 2017. While the studies Gem/Cis, Ro96 and RC57 included both locally advanced and metastatic pancreatic cancer, the two latest trials ACCEPT and RASH only recruited patients with metastatic disease. Three trials had a phase II design, 2 studies were phase III protocols (for details see Table 1). In all protocols, a life expectancy of at least 3 months (based on the assessment of the treating physician) had been requested as a trial inclusion criterion.

Individual patient data from 912 patients were included in a combined database, time-to-event endpoints were analyzed by the Kaplan–Meier method, differences between groups were compared using the log-rank test. Chi-squared nonparametric statistics were employed to analyze categorical data and determine if any significant associations or differences existed among the variables. For statistical analysis, the SPSS software package, version 29, was used.

## Results

In the 912 patients included in this pooled analysis, median age was 63 years (range 24–89 years), 59% of the patients were male and 83% had metastatic disease at study entry. Thirty-two percent of the patients analyzed had an ECOG performance status of 0, 54% an ECOG of 1 and 10% an ECOG of 2. The median OS was estimated with 7.1 months (95% CI 6.5–7.6 months) in the entire population of study patients and is 9.0 months (95% CI 8.4–9.6 months) for all patients with a survival ≥ 3 months. 48% of patients included in the AIO trials received any kind of second-line therapy after failure of first-line study treatment.

The detailed results of the 3-month survival analysis are summarized in Table 2: in the overall dataset, 21% of the patients died before month 3 after trial inclusion, with the numerically highest (26%) rate in the phase III RC57 (AIO-PK0104) study and the lowest (15%) in the RASH study. Further subgroup analysis showed a significant correlation of the 3-month survival rate with the use of subsequent treatment after failure of first-line therapy (*p* < 0.001) and ECOG performance status (*p* < 0.001). No association with 3-month survival was found for gender (*p* = 0.972), age (*p* = 0.227), and primary tumor localization in the pancreas (*p* = 0.058).

**Table 2:** Three-month OS rate in the pooled dataset as well as in the included trials (*n* = 887)

Study	OS < 3 months, *n* [%]	OS ≥ 3 months, *n* [%]
Pooled dataset	185 [20.9]	702 [79.1]
Gem/Cis	36 [18.9]	154 [81.1]
Ro96	32 [19.5]	132 [80.5]
Rc57	72 [26.3]	202 [73.7]
ACCEPT	24 [20.9]	91 [79.1]
RASH	21 [14.6]	123 [85.4]

*OS* overall survival

## Discussion

Conducting clinical trials in advanced pancreatic cancer remains a challenge, with a high proportion of negative studies and a highly vulnerable, poor-prognosis patient population. The definition of trial inclusion criteria is essential for the trial population and in the end of course also for the efficacy and safety results of a study. A good example in this context is the PRODIGE/ACCORD-11/0402 trial that investigated the use of the intensive 4-drug regimen FOLFIRINOX in metastatic pancreatic cancer: this study had restrictive inclusion criteria on host factors like performance status, organ function (e.g., liver) and co-morbidities [2]. Despite these rather “objective” inclusion criteria, many (if not nearly all) trials investigating systemic therapy in advanced pancreatic cancer during the last two decades also included a life expectancy of at least 3 months as a criterion for study entry: a fact that also is true for all our 5 AIO studies analyzed here [4–8]. However, as the current pooled analysis shows, a significant proportion of patients (21%) treated in these five protocols did in fact not survive more than 3 months after study entry. The 3-month survival rate was the highest (86%) in the RASH study (Table 2), which of interest used the same inclusion criteria as the PRODIGE/ACCORD-11/0402 study, as it also investigated the use of FOLFIRINOX (in patients not developing skin rash after a 4-week lead-line treatment with gemcitabine + erlotinib) [7].

One might conclude from these data that a strict selection of patients based on organ function, performance status, and co-morbidities better selects poor-prognosis patients than the sole assessment of expected prognosis by the treating oncologist. In a cross-trial comparison approach estimated from the published Kaplan–Meier curves from PRODIGE/ACCORD-11/0402 and MPACT, an early death rate of about 10–15% occurred during the first 3 months after randomization in these pivotal phase III trials as well [2, 3]. A proportion of approximately 20% of patients not alive 3 months after study entry was also reported in the international phase III NAPOLI-3 trial, that was presented recently at the 2023 annual meeting of the American Society of Clinical Oncology (ASCO) and compared a novel regimen called NALIRIFOX with standard gemcitabine and nab-paclitaxel [10].

When reflecting on these—at least in our opinion—clinically relevant data, one must keep in mind that a transfer of data from controlled clinical trials in advanced pancreatic cancer to a patient population that we see in the clinical routine may have several limitations. In the meanwhile it is quite evident that a significant proportion of European patients with advanced pancreatic cancer may not receive tumor-specific treatment at all and that survival in unselected, population-based registries often is reported to be very poor (in the range of 2–3 months) for patients newly diagnosed with metastatic pancreatic cancer [11–15]. Thus, the oncological community should make every effort to increase the proportion of patients with pancreatic cancer who receive effective tumor-specific treatment, enhance the recruitment for clinical trials and select patients for trials not only under aspects of drug approval but also for post-approval generalizability of the data real-word patients [16].

In the current literature, only limited studies and articles address the question whether the 3-month life expectancy is a valid inclusion criterion for clinical trials, especially in the phase II and phase III setting [9]. Based on an own literature search, the authors did not find publications on this topic for pancreatic cancer up to now; however, some evidence is available from other gastrointestinal malignancies like colorectal cancer [17, 18].

In conclusion, the authors believe that—also based on our data reported here—the common standard inclusion criterion of “3-month life expectancy” for clinical trials in pancreatic cancer should be discussed very critically and a removal of this criterion on the list of trial participation criteria seem reasonable for future studies. A selection of patients should instead be performed using relevant host factors (like organ function, performance status, and co-morbidities) and ideally also novel parameters that reflect tumor biology—while this may, on the other hand, may limit the generalizability of trial results.

## Data Availability

Available from the corresponding author upon reasonable request.
